# Supplementary figures and images for: Genome-wide analysis of bZIP transcription factors and their expression patterns in response to methyl jasmonate and low-temperature stresses in Platycodon grandiflorus
Source: PeerJ. 2024 Apr 30;12:e17371. doi: 10.7717/peerj.17371 (PMC11067905; doi:10.7717/peerj.17371)

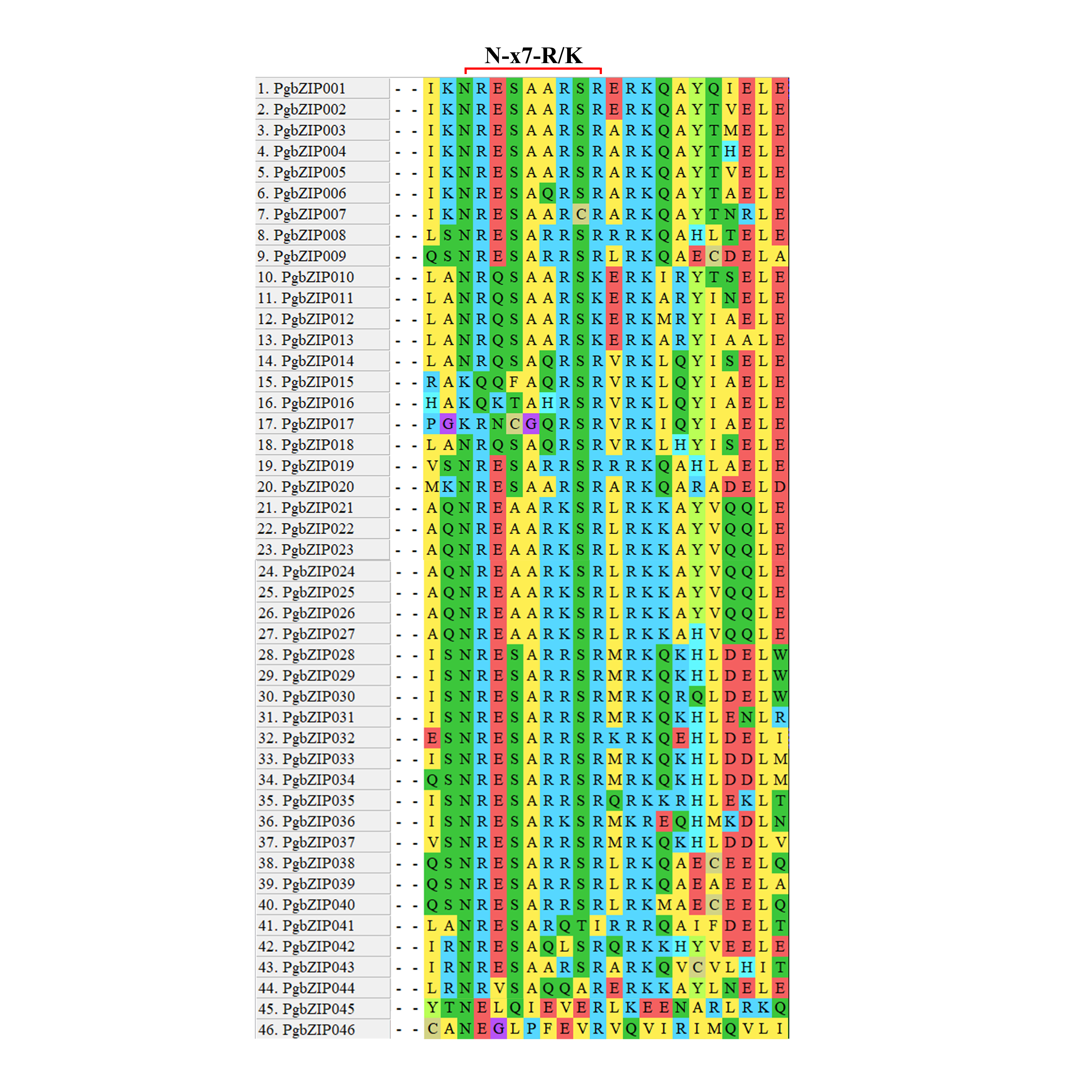

Supplement: Supplemental Information 1 [file peerj-12-17371-s001.jpg]
